# Perception of health risks of electromagnetic fields by MRI radiographers and airport security officers compared to the general Dutch working population: a cross sectional analysis

**DOI:** 10.1186/1476-069X-10-95

**Published:** 2011-11-09

**Authors:** Diana van Dongen, Tjabe Smid, Daniëlle RM Timmermans

**Affiliations:** 1 Department of Public and Occupational Health, EMGO Institute for Health and Care Research, VU University Medical Center, PO Box 7057, 1007 MB Amsterdam, The Netherlands; 2 KLM Health Services, Schiphol, Netherlands

**Keywords:** concerns, occupational exposure, risk perception, MRI radiographers, security officers

## Abstract

**Background:**

The amount of exposure to electromagnetic fields (EMF) at work is mainly determined by an individual's occupation and may differ from exposure at home. It is, however, unknown how different occupational groups perceive possible adverse health effects of EMF.

**Methods:**

Three occupational groups, the general Dutch working population (n = 567), airport security officers who work with metal detectors (n = 106), and MRI radiographers who work with MRI (n = 193), were compared on perceived risk of and positive and negative feelings towards EMF in general and of different EMF sources, and health concerns by using analyses of variances. Data were collected via an internet survey.

**Results:**

Overall, MRI radiographers had a lower perceived risk, felt less negative, and more positive towards EMF and different sources of EMF than the general working population and the security officers. For security officers, feeling more positive about EMF was not significantly related to perceived risk of EMF in general or EMF of domestic sources. Feeling positive about a source did not generalize to a lower perceived risk, while negative feelings were stronger related to perceived risk. MRI radiographers had fewer health concerns regarding EMF than the other two groups, although they considered it more likely that EMF could cause physical complaints.

**Conclusions:**

These data show that although differences in occupation appear to be reflected in different perceptions of EMF, the level of occupational exposure to EMF as such does not predict the perceived health risk of EMF.

## Background

In Europe, a debate is ongoing on exposure limits to protect workers from exposure to electromagnetic fields (EMF) [1]. The European Parliament and Council aimed to adopt a Directive which would protect workers who are exposed in the course of their work from known short-term adverse health effects [2]. Magnetic Resonance Imaging (MRI) researchers and many patients groups protested against the Directive and were given a four year reprieve to find a solution, while this would have serious consequences for clinical and research uses of MRI equipment, a device of which exposure would exceed the proposed exposure limits. Little is known about how MRI radiographers perceive possible health risk of EMF, or other workers who are occupationally exposed to EMF. In the last decade, increased mobile phone use and the consequent growing number of mobile phone base stations has led to a raise in concerns about health effects of EMF among citizens [3,4]. Studies on adverse health effects of extreme low frequencies and of radiofrequencies, such as the development of brain tumors and childhood leukemia in the long-term, and short-term health effects, like headaches and dizziness, showed scientific controversy [5-7]. Little is known about how the working population perceives the health risk of different EMF sources, in particular those working with specific EMF-emitting equipment. A study [8] showed that during clinical imaging MRI workers can be exposed to magnetic fields exceeding the guidelines of the EU-Directive 2004/40/EC [2]. There are a few studies on occupational exposure to MRI equipment and possible health effects [9,10]. A study by de Vocht [10] showed that MRI workers reported more complaints (vertigo, metal taste and concentration problems) than X-ray workers in a manufacturing department. Hardly any studies are done on the perceived health risks of EMF among MRI workers or have been conducted on occupational sources of EMF with field strengths far below the EMF exposure limits, such as metal detectors in airports, public buildings and shops.

People's subjective judgement of the probability and severity of adverse health effects of different EMF sources differ from the health risk as perceived by experts [11,12]. EMF is comprised by different ranges of frequencies, such as radiofrequency (RF), extremely low frequency (ELF), as well as sub ELF (static field), which have different properties and interact differently with the human body [13]. High voltage power lines and MRI equipment radiate ELF, while mobile phone base stations and mobile phones radiate RF. In general, lay people are not aware of this technological difference, but do rate different EMF sources differently regarding the risk for health. Factors such as not being observable, uncontrollability, and involuntariness of exposure to the hazard influence people's perception of adverse health risk from a hazard [12]. Health risk of EMF may be perceived differently depending on a worker's knowledge and the voluntariness of exposure. In addition, people's feelings influence their decision-making and play a prominent role in the risk perception [14-17]. When people see the benefit of a technology and have a positive feeling towards it, they estimate the risk lower than when they have a negative or less positive feeling towards the technology [14]. How different occupational groups perceive the possible risk of different EMF sources is unknown.

We compared three different occupational groups: the general Dutch working population; security officers, who work regularly with a source of EMF with field strengths far below EMF exposure limits; and MRI radiographers, who work regularly with a source of EMF with field strengths close to EMF exposure limits and a strong static magnetic field. We compared these three groups on the perception of risk, and negative and positive feeling towards different EMF sources and towards EMF in general. Three different types of sources were compared: domestic, occupational, and environmental, differing in voluntariness of exposure. Domestic sources are sources people choose to have or use (voluntary), for example a mobile phone, a cordless phone (digital enhanced cordless telecommunication (DECT)), and a microwave oven (though it is recognised that concerns about each of these domestic sources can be very different). Occupational sources are sources specifically used at work (semi-voluntary), such as MRI equipment and a metal detector. Environmental sources are sources not owned personally by individuals and to which people are involuntarily exposed, such as power lines and mobile phone base stations. We also compared the occupational groups on health concerns.

With this study we wanted to find answers to the following questions: (1) Do the occupational groups differ on perceived risk of and feelings towards EMF in general and of different EMF sources, and if so to what extent?; (2) What is the relationship between negative and positive feeling with perceived risk of EMF in general and of different EMF sources of the occupational groups?; (3) Do the occupational groups differ on health concerns with respect to EMF and different EMF sources, and if so to what extent?

## Methods

### Participants

Three groups were studied: Group 1 consisted of people of the general Dutch working population; group 2 consisted of airport security officers who regularly work with a source of EMF with field strengths far below EMF exposure limits (metal detector); and group 3 consisted of MRI radiographers who regularly work with a source of EMF with field strengths close to EMF exposure limits (MRI equipment). The working population was approached via an invitation in an online consumer panel (20,000 members, ISO 20252 and ISO 26362). The panel consisted of a representative sample of the adult Dutch population. The sample was stratified on demographic features (age, gender, educational level and area of residence) to ensure that the sample is an accurate representation of the population. Panel members were not invited to participate if they had cooperated in a previous study on EMF earlier that year. Participants received points for cooperation, which could be exchanged for gifts. Within a week 1009 participants had filled out the questionnaire. Of the 1009 participants, 57% were employed and included in this study. Nine participants were excluded because they worked with EMF sources with exposures above Action Level [13], the exposure level at which personal reduction is obligatory. This working population was equal on age, gender and educational level to the Dutch working population according to the CBS (Statistics Netherlands).

The security officers were recruited via a security company at Schiphol/Amsterdam airport. They were approached in their break by one of the researchers. They were asked to participate in an anonymous, online study on EMF sources in daily life and at work. In exchange for their e-mail address they received a small incentive, which resulted in 217 officers signing up.

MRI radiographers were recruited via a call from the Dutch Society for Medical Imaging and Radiotherapy (NVMBR) through an advertisement in their monthly journal and through contact by a NVMBR representative in most Dutch hospitals. The call described that the study was about the use of EMF in daily life and at work. MRI radiographers were asked to send their e-mail address. To this call 344 MRI radiographers from 36 hospitals responded.

Both security officers and MRI radiographers who had signed up received an e-mail with a link to the questionnaire. They received a first reminder after a week and a second one after two weeks. The response rate of the security officers was 49%. The response rate of the MRI radiographers was 56%. The security officers received, additionally to the small incentive they got for their e-mail address, company points after filling out the questionnaire, which they could exchange for gifts. The MRI radiographers received a small incentive.

### Outcome measures

The items used in this study were selected from a larger questionnaire on risk perception and EMF. In the introduction of the questionnaire, it was explained to participants that the questions involved EMF, but no description of EMF was provided. The concepts measured were risk perception of EMF in general, risk perception of different EMF sources, positive and negative feelings towards EMF in general and of different EMF sources, and concerns about health effects of EMF.

#### Perceived risk of and feelings towards EMF in general and different EMF sources

Risk perception of EMF was measured by asking the participants how dangerous they thought EMF was ("To what extent do you think EMF is dangerous?", rated on a 5-point scale from 'very harmless' to 'very dangerous'). Negative and positive feeling towards EMF were measured by asking the participants how frightening they thought EMF was ("When I think of EMF I think it is frightening", rated on a 7-point scale from 'not at all' to 'very') and how positive their feeling was towards EMF ("When I think of EMF I think it is positive", rated on a 7-point scale from 'not at all' to 'very').

To measure the perceived risk of the different EMF sources, participants were asked to rate questions on risk perception ("To what extent do you think this technology is a health risk?"), positive feeling ("To what extent do you have a positive feeling about this technology?") and negative feeling ("To what extent do you get an unsafe feeling of this technology?") on a 5-point scale from 'not at all' to 'very much'.

Eight different EMF sources were selected based on their different properties. Three types were used: domestic, occupational, and environmental. Domestic sources included commonly used equipment, namely a mobile phone, a microwave oven, and a cordless phone (DECT). Occupational sources included the MRI and a metal detector. Environmental sources included a GSM base station, a UMTS base station and power lines. Both UMTS and GSM base station were included while previous focus group interviews (data not published) showed that people were more concerned about the new UMTS base station, than the older GSM base station. To compare these three types of EMF sources, a group mean of risk perception was calculated for the domestic sources and the environmental sources. Risk perception of a DECT, a microwave oven, and a mobile phone were summed into domestic source (α = .88). Risk perception of power lines, a GSM base station, and an UMTS base station were summed up for environmental source (α = .93). The metal detector and the MRI were not combined for the occupational source, since too many differences in risk perception were expected between these two sources.

#### Concerns about health effects of EMF

With regard to concerns about health effects, a distinction between diseases and physical complaints was made. The difference between diseases and physical complaints was explained in the text prior to the questions about the health effects. Participants were told that: "In the next part a distinction is made between physical complaints and diseases. Diseases are e.g. diabetes, flu, and malaria. Physical complaints are symptoms that are not specifically part of the disease you might have right now, e.g. dizziness, pain, and shortness of breath." Participants were asked whether they believed people could get a disease from EMF. If answered 'yes', two other questions followed: "How many people in the Netherlands do you think will get a disease caused by EMF?" (on a 6-point scale from 'less than 10' to 'more than 100,000') and "What are the chances of getting a disease of EMF yourself?" (on a 5-point scale from 'not/very low' to 'very high'). The same questions were asked about physical complaints. We assumed that participants knew that the Dutch population size is approximately 16 million, therefore, no indication of it was given with the questions.

### Data analyses

Analyses of variances were conducted to compare the three occupational groups on risk perception, and negative and positive feeling of EMF in general. The same statistical analyses were used for risk perception, and negative and positive feeling per EMF source. Repeated measures analysis of variance was used to compare differences between the occupational groups on perceived risk of the different types of sources. Between subjects factor was the occupational group and the within subjects factor the risk perception of domestic sources, metal detector, MRI, and environmental sources. Bonferroni correction was applied for post hoc comparison groups on all of the above analyses. To verify that the differences between the groups were not related to differences in educational level, Pearson correlation was used within the general working population to see if educational level influenced the other variables.

Pearson correlation was used to measure the correlations between risk perception, negative and positive feelings of EMF in general, domestic sources, metal detector, MRI, and environmental sources within each occupational group. Fisher's z was used to test whether correlations differed significantly between groups and different EMF sources.

Pearson chi-square tests were conducted to analyse differences between the three occupational groups in whether participants believed EMF could cause a disease. Pearson chi-square tests were performed per item comparing two out of the three groups with each other. To analyse how many Dutch participants were believed to have a disease caused by EMF, the question was dichotomized on the median. Pearson chi-square tests were used to compare the groups on differences regarding estimates larger than 10,000, the median. Analysis of variances was used to compare the groups on what they thought the chances were of getting a disease themselves caused by EMF. The same statistical analyses were used for physical complaints.

## Results

### Participants

The group characteristics demonstrate some differences between the groups. MRI radiographers are usually higher educated women, while the security officers are younger and their educational level was mainly medium (see Table 1). No strong significant correlations were found within the general working population between educational level and the variables perceived risk of EMF in general and of different EMF sources and feelings towards EMF in general and of different EMF sources (-.17 < r^2 ^> .15). Therefore, we can presume that the found differences between the occupational groups were not related to the difference in educational level between the groups.

**Table 1:** Group characteristics

	Working population (N = 567)	Security officers (N = 106)	MRI radiographer (N = 193)
Gender (% male)	58.2	52.8	23.3

Age (m (sd))	42 (12.5)	37 (10.9)	40 (9.4)

Education level ° (%)			
Low	24.2	5.7	0
Medium	43.4	82.1	5.2
High	32.5	12.3	94.8

° Low: primary school, lower level of secondary school, or lower vocational training. Medium: higher level of secondary school, or intermediate vocational training. High: higher vocational training or university.

### Perceived risk of and feelings towards EMF in general and of different EMF sources

Table 2 shows risk perception of and feelings towards EMF in general and of different EMF sources of the three groups. Analyses of variance showed that MRI radiographers felt significantly more positive and less negative about EMF and had a lower perceived risk of EMF than the general working population and security officers.

**Table 2:** Means and standard deviations of risk perception and feelings towards EMF in general and different sources per group

		Risk perception (5-point scale: "very harmless"-"very dangerous")			Negative feeling (5-point scale: "no"-"very much")			Positive feeling (5-point scale: "no"-"very much")		
(**m(sd))**		**Working pop**.	**Security officers**	**MRI radiogr**.	**Working pop**.	**Security officers**	**MRI radiogr**.	**Working pop**.	**Security officers**	**MRI radiogr**.

EMF in general		3.0^3 ^(0.8)	3.0^3 ^(0.7)	2.7 (0.7)	2.8^2^,^3 ^(1.0)	2.3^3 ^(1.1)	1.8 (0.9)	3.2^2^,^3 ^(1.1)	2.1 (0.9)	2.8^2 ^(0.9)

Domestic sources	Microwave oven	2.3^3 ^(1.1)	2.8^1^,^3 ^(1.1)	2.1 (0.9)	2.0 (1.0)	2.3^1^,^3 ^(1.1)	1.9 (0.9)	3.5 (1.1)	3.6 (1.1)	3.7 (0.9)
	
	Mobile phone	2.6^3 ^(1.1)	2.9^1^,^3 ^(1.2)	2.3 (0.9)	2.0 (1.0)	2.3^1^,^3 ^(1.2)	1.9 (0.9)	3.8 (1.0)	3.9 (1.0)	3.9 (0.8)
	
	DECT	2.3^3 ^(1.0)	2.5^3 ^(1.1)	2.1 (0.9)	1.9 (1.0)	2.1^1^,^3 ^(1.0)	1.8 (0.9)	3.6 (1.1)	3.5 (1.1)	3.8^1^,^2 ^(0.9)

Occupational sources	MRI	2.8^3 ^(1.1)	2.9^3 ^(1.1)	2.5 (0.9)	2.3 (1.2)	2.4^3 ^(1.2)	2.1 (1.0)	3.6 (1.2)	3.4 (1.2)	4.2^1^,^2 ^(0.9)
	
	Metal detector	2.3^3 ^(1.0)	2.8^1^,^3 ^(1.1)	2.0 (0.9)	2.0 (1.0)	2.5^1^,^3 ^(1.2)	1.8 (0.9)	3.1 (1.1)	3.5^1 ^(1.1)	3.3 (1.0)

Environmental sources	Power lines	3.0 (1.3)	3.4^1^,^3 ^(1.2)	2.8 (1.1)	2.6 (1.3)	2.9^1 ^(1.4)	2.7 (1.3)	3.0 (1.2)	2.8 (1.1)	3.1 (1.2)
	
	GSM base station	3.0^3 ^(1.2)	3.2^3 ^(1.2)	2.7 (1.0)	2.4 (1.3)	2.6 (1.3)	2.4 (1.2)	3.0 (1.1)	3.0 (1.2)	3.1 (1.1)
	
	UMTS base station	3.1^3 ^(1.2)	3.2^3 ^(1.2)	2.8 (1.1)	2.5 (1.3)	2.7 (1.3)	2.5 (1.2)	2.9 (1.1)	2.9 (1.2)	3.0 (1.1)

^1 ^p < .05, significantly higher (ANOVA) than the working population, ^2 ^p < .05, significantly higher (ANOVA) than the security officers, ^3 ^p < .05, significantly higher (ANOVA) than the MRI radiographers. With Bonferroni correction.

The MRI radiographers rated all sources of EMF as being significantly less hazardous for health (risk perception) than the other two groups, including their own occupational source, the MRI equipment.

Security officers rated almost all sources of EMF more hazardous than the other two groups, including their own occupational source, the metal detector. The largest differences between the three groups in feelings towards the different EMF sources was found for the occupational sources. MRI radiographers were most positive about the MRI equipment and significantly more than the other two groups. We also found that security officers were significantly more positive about the metal detector than the working population, but at the same time, they were also significantly more negative about the metal detector than the other two groups.

There were also differences in the risk perception of different types of EMF sources. Repeated measure analysis of variance showed an overall effect of sources (F(3, 858) = 6.97; p < .01) with all sources differing significantly from each other and with the metal detector having the lowest rating on perceived risk and environmental sources the highest. Domestic sources were overall rated significantly less hazardous for health than environmental sources.

For negative feeling towards the EMF sources, repeated measure analysis of variance also showed an overall effect of sources (F(3, 858) = 7.00; p < .01) with the lowest negative feeling for domestic sources and the highest for environmental sources. Repeated measure analysis of variance also showed an overall effect of sources for positive feeling towards different EMF sources (F(3, 858) = 8.61; p < .01). Domestic sources were rated most positive, but did not significantly differ from the MRI. Environmental sources were rated least positive.

### Relationships between perceived risk of and feelings towards EMF in general and of different EMF sources

The security officers did not differ in perceived risk of EMF from the general working population, but felt significantly less negative and less positive about EMF in general. Negative feeling correlated in all three groups significantly with perceived risk (general working population: r = .61; security officers: r = .36; MRI radiographers: r = .44, p < .05). With the exception of the security officers (r = -.12, p = .240), positive feeling and risk perception of EMF had a significantly negative correlation in the general working population (r = -.50, p < .05) and a weak but significant negative correlation in the MRI radiographers group (r = -.28, p < .05). Fisher's r to z transformation showed that for the general working population there was a stronger positive correlation between risk perception and negative feeling than for the MRI radiographers (z = 3.10, p < .01) and the security officers (z = 2.82, p < .01). Also, a stronger negative correlation between risk perception and positive feeling was found for general working population compared to the MRI radiographers (z = -3.12, p < .01) and the security officers (z = -4.00, p < .01).

In Table 3 the correlations between perceived risk of and feelings towards EMF in general and of different sources are demonstrated per group. The majority of outcome measures correlate within the groups. There were strong positive correlations between risk perception and negative feeling of the different EMF sources which were stronger than the correlations between risk perception and positive feeling. With the exception of the security officers on domestic sources, positive feeling and risk perception of the different EMF sources have significant negative correlations. Fisher's z showed that most correlations did not differ significantly between the three occupational groups. Exceptions were: the correlations of environmental sources of security officers and MRI radiographers for negative feeling (z = 2.03, p < .05) and for risk perception and positive feeling (z = -2.34, p < .05), and the correlations of metal detectors of working population and MRI radiographers for negative feeling (z = -2.39, p < .05).

**Table 3:** Correlations between risk perception and feelings of different EMF sources per occupational group.

	Negative feeling			Positive feeling		
	**Working pop**.	**Security officers**	**MRI radiogr**.	**Working pop**.	**Security officers**	**MRI radiogr**.

Risk perception domestic	.65**	64**	71**	-.35**	-.18	-.38**

Risk perception MRI	57**	55**	61**	-.21**	-.26**	-.30**

Risk perception metal detector	57**	.65**	69**	-.24**	-.36**	-.32**

Risk perception environmental	68**	62**	75**	-.36**	-.22*	-.47**

**. Correlation is significant at the 0.01 level.

*. Correlation is significant at the 0.05 level.

### Concerns about health effects of EMF

68% of all the participants believed that EMF could cause a disease and 76% believed it could cause physical complaints. There were no significant differences between beliefs about a disease and beliefs about developing physical complaints (see Table 4), with the exception of the MRI radiographers. Pearson chi-square tests showed that significantly more MRI radiographers believed that people could get physical complaints from EMF compared to the working population and security officers.

**Table 4:** Percentages and estimates of participants' health concerns per occupational group.

	Do EMF have health effects? (Number yes (%)) ^a^			How many Dutch people have health effects? (% above median)^a^			What are the chances of getting health effects yourself? (mean (SD))°		
	**Working pop**.	**Security officers**	**MRI radiogr**.	**Working pop**.	**Security officers**	**MRI radiogr**	**Working pop**.	**Security officers**	**MRI radiogr**.

Disease	397^3 ^(70%)	73 (70%)	118 (61%)	26.2^3^	35.6^3^	11	2.2^3 ^(0.8)	2.7^1^,^3 ^(1.0)	2.0 (1.0)

Physical complaints	423 (75%)	78 (74%)	161^1^,^2 ^(83%)	30.3^3^	29.5	20.5	2.3 (0.8)	2.7^3 ^(1.0)	2.2 (1.1)

^a ^Pearson chi-square test; ° 5-point scale from "not" to "very high"

Analyses of variances, ^1 ^p < .05, significantly higher than working population, ^2 ^p < .05, significantly higher than security officers, ^3 ^p < .05, significantly higher than MRI radiographers

Also, a lower percentage of the MRI radiographers had the opinion that over 10,000 Dutch people a year have physical complaints due to EMF compared to the other 2 groups. Analysis of variance showed that security officers rated the chances of getting a disease themselves as significantly higher than the general working population and the MRI radiographers, and the chances of getting physical complaints themselves significantly higher than MRI workers (see Table 4).

## Discussion

This study shows that not all occupational groups have the same risk perception of EMF and EMF sources. The MRI radiographers rated EMF and different EMF sources as less hazardous for health than did the general working population and the security officers. Ratings of the security officers did not differ much from the general working population. In line with earlier studies, all three groups rated the less voluntary and less controllable environmental sources as a higher risk for health than the more voluntary and more controllable domestic sources [3,18-21]. Negative feeling towards EMF were associated with a higher perceived risk [11]. Additionally, more than two third of the participants believed that EMF could cause adverse health effects..

This study has some limitations that need to be addressed. First, the study presents cross-sectional data and can not show the dynamic relationships between the variables. Therefore, only descriptive analyses were performed, demonstrating differences between the occupational groups. Second, the precise occupations of the general Dutch working population are unknown. We excluded participants who reported working with devices close to EMF exposure limits, but whether they work with other EMF sources, and of what category, is not known. Therefore, this group serves primarily as a control group to compare with the two specific occupational groups. Also, the exact dose and duration of EMF exposure per individual within the specific occupational groups is not known. However, it seems unlikely that these individual differences in exposure would affect the differences in perceived risk and health concerns between occupational groups to a large extent.

The two occupationally-exposed groups differed in their perception of health risk of EMF and EMF sources. MRI radiographers' perceptions of risk were lower than those of the security officers, although they work with equipment with EMF strengths closer to the exposure limit than security officers. The level of occupational EMF exposure does not seem to influence the risk perception of employees. Experience, training, as well as being more technology-orientated might affect risk perception of EMF. MRI radiographers have more education and training in their occupational EMF source - the MRI - than the security officers have in the metal detector and are probably more technology-orientated than security officers as their job is more technical. However, it should be noted that MRI radiographers are only made aware of possible health effects, apart from the risk of missiles, in training in safety precautions to avoid acute effects, such as nausea, dizziness, and metal taste in the mouth. No information is given about other possible adverse health effects. It thus seems likely that training is not the only explanation for the differences between MRI radiographers and security officers.

Security officers had an ambivalent attitude towards the metal detector, as they reported more negative as well as more positive feelings towards the metal detector. Focus group interviews, conducted prior to the present study to develop the questionnaire (data unpublished), showed that security officers are only taught that EMF is a non-ionizing radiation and that therefore, no special safety precautions are necessary. Also, most security officers are employed for less than five years, so this profession might be seen as a just in between job, which may make them less devoted to their job and less interested in what is needed for the job - the metal detector. Furthermore, the metal detector is a device to aid security officers in their work, but is less essential for their work. As proposed by the Affect Heuristic [14], some security officers may think more positively about the metal detector because it makes their job easier while at the same time they are concerned about adverse health effects. Others might think more negatively about the metal detector as a result of a lower perceived benefit and higher perceived risk of the metal detector. One reason that MRI radiographers are more positive towards the MRI, might be that the MRI is essential for the job of the MRI radiographers and therefore the benefits might be more apparent.

Participants' beliefs about EMF causing a disease, such as cancer, or causing physical complaints, such as dizziness or headache, did not differ much. This suggests that people do not take into consideration the different mechanisms causing complaints or causing disease. As MRI radiographers have a better medical education and deal with seriously ill patients on day to day basis, they might be aware of this and therefore do make a distinction herein.

Risk communication by the authorities or employers often uses technological and numerical information to explain certain risks [2,13], in order to reduce the (potential) risk perception. But anticipating on feelings and attitudes towards the risk source might be of more importance. In this study we showed that different occupationally exposed groups have different thoughts and feelings about EMF and EMF sources. When looking at the technological information, MRI radiographers can be expected to be more concerned about the EMF exposure than the security officers. But in fact MRI radiographers are little concerned, while security officers are more concerned. We suggest that employers fit (risk) communication to the thoughts and feelings of the employees towards the EMF sources and not presume that employees are not worried when working with sources far below the exposure level.

## Conclusions

Although no clear long term adverse health effects of exposure to EMF have been shown to date, occupational groups working with EMF equipment differ in their perceptions of possible risks. The strength, as opposed to the nature, of occupational exposure as such does not seem to be of major influence on the risk perception, but knowledge of EMF in general and voluntariness of exposure of different EMF sources likely is. It is important to keep this in mind when informing employees about possible risk of EMF on health.

## Abbrevarions

EMF: Electromagnetic field; MRI: Magnetic Resonance Imaging; RF: radiofrequency; ELF: extreme low frequency; DECT: digital enhanced cordless telecommunication.

## Competing interests

The authors declare that they have no competing interests.

## Authors' contributions

DvD contributed to the design of the study and questionnaires, performed the statistical analyses, and drafted the manuscript. TS contributed to the design of the study and questionnaires, helped to draft the manuscript and revised it critically for statistical and intellectual content. DT contributed to the design of the study and questionnaires, helped to draft the manuscript and revised it critically for statistical and intellectual content. All authors read and approved the final manuscript.
